# An Interesting Question in Auscultation

**Published:** 1883-01

**Authors:** P. W. Van Peyma


### Clinical Reports.

### An Interesting Question in Auscultation.

By P. W. Van Peyma, M. D.

Mr. B., a blacksmith, in the neighborhood of 36 years of age, has for a period of considerably over a year been a consumptive. The disease is, at present, nearly limited to the left lung. The upper portion of the right lung is, however, not quite free from tubercular trouble. A considerable cavity has formed in the left lung anteriorly. Upon examination by auscultation, a few days since, a distinct tinkling sound was heard in the neighborhood of the left nipple, synchronous and additional to the normal cardiac murmurs. This tinkling was more readily heard by direct auscultation, but was also transmitted through Cammans' stethoscope, being readily heard by the patient himself. The sound was, in my opinion, due to the impulse of the heart upon the walls of the cavity. While I have not given the subject any special research, I do not remember having seen it noticed in medical literature.

